# How are medical groups identified as high-performing? The effect of different approaches to classification of performance

**DOI:** 10.1186/s12913-019-4293-9

**Published:** 2019-07-18

**Authors:** Sangeeta C. Ahluwalia, Cheryl L. Damberg, Ann Haas, Paul G. Shekelle

**Affiliations:** 1 RAND Corporation, 1776 Main Street, Santa Monica, CA 91403 USA; 2 UCLA Fielding School of Public Health, Los Angeles, CA USA; 3 RAND Corporation, Pittsburgh, PA USA; 4 VA West Los Angeles Medical Center, Los Angeles, CA USA

**Keywords:** Performance measurement, Quality, Health system

## Abstract

**Background:**

Payers and policy makers across the international healthcare market are increasingly using publicly available summary measures to designate providers as “high-performing”, but no consistently-applied approach exists to identifying high performers. This paper uses publicly available data to examine how different classification approaches influence which providers are designated as “high-performers”.

**Methods:**

We conducted a quantitative analysis of cross-sectional publicly-available performance data in the U.S. We used 2014 Minnesota Community Measurement data from 58 medical groups to classify performance across 4 domains: quality (two process measures of cancer screening and 2 composite measures of chronic disease management), total cost of care, access (a composite CAHPS measure), and patient experience (3 CAHPS measures). We classified medical groups based on performance using either relative thresholds or absolute values of performance on all included measures.

**Results:**

Using relative thresholds, none of the 58 medical groups achieved performance in the top 25% or 35% in all 4 performance domains. A relative threshold of 40% was needed before one group was classified as high-performing in all 4 domains. Using absolute threshold values, two medical groups were classified as high-performing across all 4 domains. In both approaches, designating “high performance” using fewer domains led to more groups designated as high-performers, though there was little to moderate concordance across identified “high-performing” groups.

**Conclusions:**

Classification of medical groups as high performing is sensitive to the domains of performance included, the classification approach, and choice of threshold. With increasing focus on achieving high performance in healthcare delivery, the absence of a consistently-applied approach to identify high performers impedes efforts to reliably compare, select and reward high-performing providers.

## Background

Improving the performance of healthcare providers (i.e., healthcare delivery systems, hospitals, and medical groups) is a principal health policy goal across international healthcare markets [1–5]. Payers and policy makers are applying a variety of levers to stimulate performance improvement by healthcare providers including public reporting of performance and recognition (e.g., CMS Star ratings in the U.S. or the UK’s National Health Service’s star ratings and Quality Outcomes Framework and financial incentives to providers designated as “high performing” [1–3, 6–12]. Pay-for-performance programs are widely used in the U.S. by public and private insurers and increasingly used in Canada, Australia, U.K, and other European countries, and even in some countries in the developing world [5, 13–15]. Patients are encouraged to choose higher performing providers using publicly available scorecards that rate and classify provider performance.

Designating a provider as “high-performing” requires an agreed-upon definition of high performance with standard decision rules and performance measures. Our recent systematic review of definitions of high performance found no consistent definition of what constitutes a high-performing provider or healthcare system [16]. Wide variation exists in how payers and researchers designate “high-performing” providers, applying different performance domains (e.g., quality, cost, access, patient experience) and types of measures (e.g., individual vs. composite measures) within those domains, and using varying thresholds. While situations may exist where focusing on one or two aspects of performance is important, designating providers as “high-performing” based on only certain performance domains (e.g., clinical quality) risks identifying providers as “high-performing” despite poor performance in other important domains (e.g., patient experience, cost). As consumers are being encouraged and even financially incentivized to obtain all or most of their care within a single health system or a medical group, it is reasonable for consumers to expect that the system or medical group they choose should be high performing across multiple dimensions of performance. Similarly, the use of individual versus composite measures has implications for how stakeholders perceive and understand high-performance [17]. Individual measures can characterize performance within single domains and identify specific processes for improvement within groups, while composite measures combine different aspects of performance and facilitate benchmarking across groups. While the merits of each of these approaches can be argued and improved upon, consumers, payers, and policy makers are typically limited to the domains and measures included in existing publicly available data for assessing provider performance and selecting or rewarding the “high-performers”. It is important to understand – through the data currently available to stakeholders – the extent to which different applications of the definition and measurement of performance impact if and how groups are identified as high-performing.

In the current study, we sought to understand the real-life implications of using different performance domains and classification approaches on designating providers as high-performers. Specifically, we assessed existing, publicly-reported provider performance on four commonly-used performance domains identified in our prior review [16] - quality, cost, access, and patient experience - and applied two different classification approaches, relative and absolute performance thresholds, to test whether different approaches resulted in different providers being designated as high-performers.

## Methods

### Data source

We used publicly available performance data from the 2014 Minnesota Community Measurement (MNCM) Health Care Quality Report (http://mncm.org/health-care-quality-report/), which reports comparative medical group performance data. The MNCM report is used by providers, payers, employers, and policymakers to guide improvement and investment efforts and by consumers as guidance for selecting among providers [18]. The medical groups in MNCM data include any combination of primary, specialty, or multi-specialty provider organizations operating under the same tax identification number. Measures included in the MNCM data were chosen by a multi-stakeholder group in Minnesota to address gaps in performance and stimulate improvement efforts [18].

### Measures

Drawing upon the Institute of Medicine’s (IOM) conceptual framework for a twenty-first century health system that is safe, effective, patient-centered, timely, efficient, and equitable [19], we examined medical group performance across 4 of the 6 IOM performance domains, that were also identified in our prior review [16]: quality, total cost of care, access, and patient experience. There were no available measures in our dataset to assess performance in the IOM domains of safety and equity.

For each of the four performance domains, we applied an “all-or-none” approach to defining high-performance [20, 21], wherein a medical group had to be high-performing on all measures within a domain, thus requiring the medical group to report on all selected measures. To maximize the number of medical groups in our sample given this “all-or-none” approach, and to ensure we were including similar types of medical groups for comparison, we selected the subset of measures within each domain reported by the largest number of medical groups, and excluded specialized measures reported by only a small number of groups, e.g., primary C-section rates (See Appendix for a full list of all measures available in the MNCN data). The included measures address ambulatory care provided by both primary care physicians and specialists and reflect commonly targeted measures for ambulatory care improvement:

- Quality: We selected 4 measures to represent the quality domain: 1) colorectal cancer screening; 2) breast cancer screening; 3) optimal diabetes care composite; 4) optimal vascular care composite.
- Cost: The National Quality Forum-endorsed total cost of care measure, which represents the average cost of care per member per month for all patients within each medical group. The methodology [22].
- Access: A composite measure from the Consumer Assessment of Healthcare Providers and Systems – Clinician and Group (CG-CAHPS) survey that assesses the availability of appointments, access to routine and urgent care, and information when needed over the past year.
- Patient Experience: We used 3 CG-CAHPS measures: courteous and helpful staff, how well providers communicate, and providers with the most positive rating.

While MNCM reports some measures at both the clinic and the medical group level, our analysis focused on the medical group as the unit of analysis because more measures of performance were reported at this level.

### Analytic sample

Of the 240 medical groups in the MNCM database, 78 reported data on all four selected quality measures (i.e., optimal diabetes care, optimal vascular care, colorectal cancer screening and breast cancer screening). Of the 78 medical groups reporting all four quality measures, 67 (86%) also reported cost data, and of these, 58 (74%) also reported the selected CAHPS measures on access and patient experience. Our final analytic sample includes 58 general medical groups reporting all selected measures across the 4 domains.

We used an annually published list of the Top 25 medical groups in Minnesota by revenue to assess the generalizability of our sample. Of the Top 25 medical groups in Minnesota by revenue in 2015 [23], 16 provided a broad range of adult medical care services (e.g., primary care, multi-specialty), while 9 provided single specialty care only (e.g., dental, senior home care, pediatrics). Of the 16 groups providing adult medical care (i.e., the groups most relevant to our study), 14 (88%) reported data across all 4 performance domains of interest and are included in our analytic sample. Thus, our sample includes almost all the largest general medical groups in Minnesota in terms of revenue; i.e., the medical groups likely to be where most Minnesotans receive their care.

Comparing included and non-included groups showed that about half of the 240 groups did not report a measure of interest. For example, 127 groups did not report the diabetes composite measure and 106 groups did not report the breast cancer screening measure. The primary reasons for not reporting were that the measure did not apply to the medical group’s patient population (e.g., a pediatric group or an orthopedic group) or the sample size of patients was too small to generate a stable estimate. Included groups performed about 10% better than non-included groups on each reported quality measure (example: median vascular composite measure scores for included versus non-included groups was 62.3% vs 59.3%) and there was a narrower distribution of scores within each measure among included groups. Included groups also were about 5% higher in cost.

### Analyses

We pre-specified the conceptual methods for our analyses. We classified medical groups as high performing using two threshold approaches commonly used in practice to classify providers: 1) relative value thresholds, where groups are ranked by performance relative to each other (e.g., top 25%, top 35%) and 2) absolute value thresholds, where groups are ranked according to pre-set or objective standards (e.g. scores above 75%, scores above 90%). Both approaches have strengths and weaknesses [24, 25].

#### Relative value threshold approach

We first ordered each medical group according to its performance in each of the four domains, and for those domains with multiple measures, on each measure within domain. For example, using a top quartile relative value threshold, a medical group had to be in the top 25% of performance for each of the four measures comprising the quality domain to be classified as performing in the top quartile for quality. To be a high performer for the cost domain, a medical group would have to perform in the lowest quartile of average costs per member per month. We tested numerous relative threshold values: top 25%, top 35%, top 40%, top 50%.

#### Absolute value threshold approach

We identified absolute score thresholds for each performance measure. Initial attempts at using a stringent absolute score threshold such as 90% (the equivalent of an “A” grade) or 80% (a “B” grade) for all measures found no medical groups would be classified as high performing. Conversely, setting the absolute threshold low enough (e.g., 50%) such that some groups would be classified as high performing on the most difficult to attain measure (the composite diabetes measure) meant that most groups were high performing on all other measures. Choosing a 50% absolute threshold for all domains would be tantamount to distinguishing medical groups based solely on their diabetes care and has limited face validity, as consumers and policymakers reasonably expect high performance to mean more than an “F” grade. We therefore set 66.6% as an initial absolute score threshold for the quality measures and 80% for the access and patient experience measures. From this initial threshold, we adjusted within each domain to avoid situations where almost all or no groups were high performing on any individual measure, resulting in the following absolute value thresholds:

- Quality – The absolute score threshold for diabetes care was set at > 0.50, for vascular care and colorectal cancer screening at > 0.66, and for breast cancer screening at > 0.75.
- Patient Experience: The absolute score threshold for each of the 3 CAHPS metrics was set at > 0.80.
- Access: The absolute score threshold for the CAHPS composite measure was set at > 0.60.

Using the lowest quartile of costs (< $420 average monthly cost of care per patient) no groups would be classified as high performing (while being high performing in other domains); therefore, we selected the 50-percentile (≤$457 average monthly cost of care per patient) as the absolute value threshold. This value represents the bottom half of all medical groups, and we judged that being able to deliver high performance on the domains of quality, patient experience and access using the above absolute value thresholds while keeping costs at or below the average had face validity as being “high performing”.

When we assessed performance across multiple domains, we only assessed combinations of domains that included quality, as this is by far the most common domain included in existing operational multi-domain definitions of performance [16].

## Results

Among the 58 medical groups, the median number of clinics in each group was three, and the median number of physicians was 34.

### Relative threshold classification approach

Based on a top 10, 25%, or 35% threshold approach, no medical groups were identified as high performing across all four performance domains. A single medical group was identified using a top 40% threshold and a second medical group was identified using a top 50% threshold (Table 1).

**Table 1:** Effect of different relative and absolute value classification methods on classification of medical groups as high performing

Threshold for high performing	Number of groups classified as high performing
Relative value method	
Top 10% in all domains	0
Top 25% in all domains	0
Top 35% in all domains	0
Top 40% in all domains	1
Top 50% in all domains	2
Absolute value method	
Achieving 90% on all measures	0
Achieving 80% on all measures	0
Achieving 70% on all measures	0
Achieving 60% on all measures	3
Achieving 50% on all measures	16

**Fig. 1:**
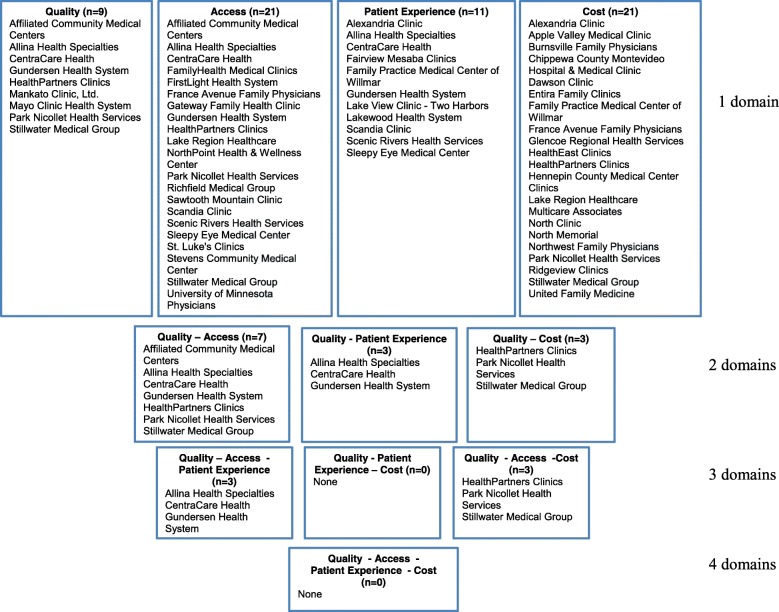
Effect of using different domains with the top 35% relative value method to define performance

Examining relative performance across combinations of three of the four performance domains, several medical groups were identified applying a top 35% threshold; however, there was little concordance in which medical groups were classified as high performers across the different combinations of performance domains (Fig. 1). For example, CentraCare Health and Gundersen performed in the top 35% for quality, access, and patient experience but not when measured across quality, access, and cost. Instead, a different set of groups – Affiliated, Health Partners and Stillwater – performed in the top 35% of quality, access, and cost (Park Nicollet and Allina performed in the top 35% for both combinations of the three domains). The difference in a medical group’s classification as high performing when exchanging a single domain (e.g., patient experience and cost) could be slight and with little implications for practice, (e.g., one group had patient experience measures ranking 12th, 22nd, and 29th, and another group ranked 22rd in cost, but ranking 21st for all measures within a domain would have been sufficient to be designated within the top 35% of performance) or it could be large and have important practice implications (one group ranked in the bottom 25% for patient experience, another group ranked 57th in cost, meaning it was the second-most-expensive group).

More groups were classified as high performers at the top 35% threshold across various combinations of performance domains when only assessed on two of the four performance domains; however, concordance in the groups identified as higher performers across different combinations of performance domains was limited.

### Absolute value classification approach

As expected, using uniform absolute value thresholds across all domains did not result in useful identification of high-performing groups. No groups were identified with thresholds of 90, 80% or 70%, only 3 groups were designated high performing at a threshold of 60%, and a threshold of 50% is roughly equivalent to distinguishing between groups solely on the basis of the diabetes composite measure (Table 1).

**Fig. 2:**
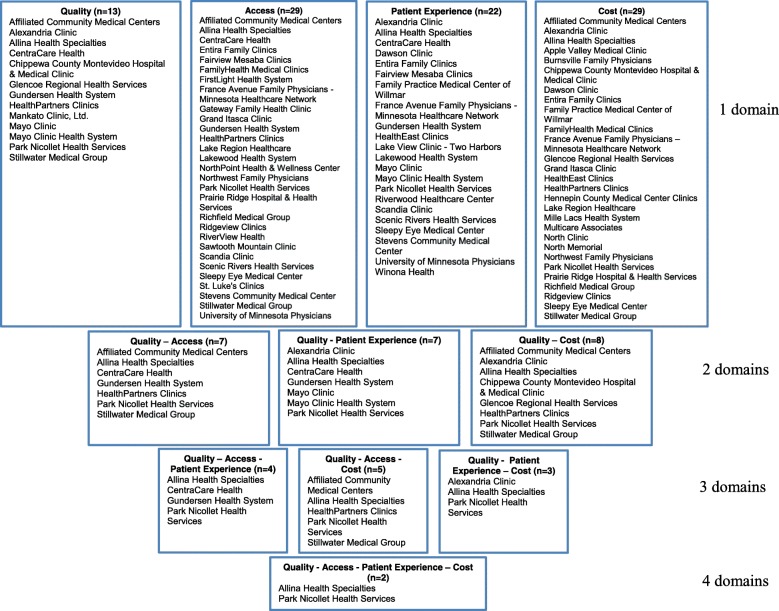
Effect of using different domains with the absolute value method to define medical group performance

Using variable absolute value thresholds for each of the metrics identified many more groups as high performers in the individual domains. For example, nearly 40% of the groups were identified as high performers in the domain of Patient Experience, and half of the groups were identified as high performers in the domains of Access and Cost. Two medical groups were identified as high performing across all four domains (Fig. 2). Similar to the relative value approach, more groups were classified as high performers with combinations of two performance domains rather than three or four domains of performance. For example, when assessing performance in quality and cost, eight medical groups were identified as high-performers, but only five of those medical groups continued to be classified as high-performers when adding the access domain.

There was only moderate concordance across combinations of performance domains in which groups were designated as high performing. For example, there were seven medical groups identified as high-performing for both quality and patient experience, but only three of those groups were also identified as high-performing for quality and cost. Five other medical groups met the threshold for high-performance in quality and cost, but only two of them met the criteria for high-performing in quality and access. As in the relative value approach, the reasons for the different groups being identified in one combination of domains but not another were in some cases small (one medical group had a score for access that was 58.5%, where 60% was needed to be classified as high performing) and in some cases very large (one group had an average cost of $823 when the threshold was $457).

## Discussion

The aim of our study was to understand how different definitional and measurement approaches to classifying performance affects which medical groups are identified as high-performing. We found the classification of medical groups as high-performing is highly sensitive to 1) the performance domains included and 2) the thresholds used within each of the domains to define performance as “high”. Regardless of threshold approach used, very few Minnesota medical groups performed in the top 50% of the distribution when assessed across all measures and no groups performed in the top tertile of all four domains. Our study also finds fewer medical groups were identified as high-performing when more domains are used to evaluate performance.

We used publicly available data currently used by consumers and payers to understand the “real-life” implications of different classification approaches. As such, we are subject to the limitations of the data we used. For example, we were limited to the number and type of measures collected and reported within the MNCM dataset. As with most measurement schemes, the clinical measures represent a fraction of all care provided, although the clinical areas measured affect a substantial portion of patients and the measures of patient experience and cost of care encompass the entire medical group’s patient population. MNCM does not assess performance in the domains of safety and equity; thus, we were only able to assess performance in the commonly-used domains of quality, cost, access, and patient experience.

Another limitation of the MNCM group-level performance data is the inability to examine the extent to which differences in case mix may influence performance, particularly for social risk factors, a concern that has been raised by a number of bodies [26–29]. To examine this would require person-level data which were not available. We note that the CAHPS measures and Total Cost of Care measures are adjusted for differences in the patients across groups, whereas the clinical measures, in keeping with National Committee for Quality Assurance (NCQA)-Healthcare Effectiveness Data and Information Set (HEDIS) measure specifications, are not. While not the aim of our study, future work might examine ways to improve performance measurement to account for differences in patient characteristics. Finally, while there may be some measurement error inherent in the different measures included in the MNCM dataset, the MNCM imposes denominator thresholds (e.g., NCQA-HEDIS minimum reporting thresholds) to ensure that the estimates of performance are reliable enough to facilitate the ability to discriminate provider performance.

The absence of a consistent approach to measuring and classifying “high-performance” has practical implications beyond our study; for example, the CMS Star Ratings program designates high performance using a clustering algorithm based on relative thresholds while the Integrated Healthcare Association (IHA) uses an absolute threshold of 50% to designate high performance [30]. The same medical groups selected for high performance in one program may not similarly be designated in another program. This creates potential confusion for consumers and sends conflicting messages to the providers being evaluated about what constitutes high performance.

A key measurement challenge facing program sponsors when benchmarking performance is how to set meaningful thresholds for classifying high-performing providers. Absolute value thresholds [24] have the distinct benefit of holding providers to an external and objective standard (similar to the “A”, “B”, “C” grades given in school) and allowing providers to target investments in improvements with specific aims. Our work showed that setting a high standard such as a score of 90% (an “A” grade) identified no groups as high-performing and would leave patients and payers with no high-performing provider options to choose from and reward. Applying a lower standard such as 50% (an “F” grade) identified most groups as high-performing on all performance measures except one, leaving patients and payers with too many indistinguishable options. In contrast, relative thresholds provide strong improvement incentives because there is no absolute level at which reward and a designation of high performer is guaranteed. Relative thresholds also allow patients to compare and select providers on the basis of average performance which may be more useful to consumers. However, relative thresholds risk rewarding poor performance when the distribution of performance is low. Whether consumers should be told there are no high-performing providers to choose from (in the case of an absolute value approach where no one earns an A grade), or that they can choose from among the “top of the pack” providers whose actual performance might be low is a dilemma payers and policymakers continue to struggle with.

Our study has some methodological limitations. We used data from a single state which may limit the generalizability of our results; however, the variation in performance on individual measures in Minnesota is consistent with variations in and levels of performance seen in data from other studies [31–33]. Although not all Minnesota medical groups were represented in our study due to the “all-or-none” approach to selecting groups for inclusion, we included nearly all major multi-specialty medical groups operating within Minnesota, supporting the representativeness of our sample and increasing the generalizability of our findings. We were limited to commonly measured domains and measures of performance; however, increasing the number of measures within each domain or the number of domains used to define performance would likely only increase the variability in how groups are classified as high-performing. Lastly, to the degree chance plays a role in determining a provider’s performance score on any particular measure in the MNCM dataset, that contribution of chance is incorporated into our results.

## Conclusion

As health care markets increasingly embrace value-based purchasing to stimulate improvement in quality and costs, a major impediment to achieving these goals is the differing approaches used to classify providers as high-performing. Our results show that differences in how “high performing” is defined may result in completely different providers being so designated, even when using the same performance measures, creating confusion for providers in how to respond. To enable common identification of high performers for use in provider payment and recognition and provider selection by consumers, an agreed-upon standard definition of high performance and approach to measurement is needed.

## Data Availability

We used publicly available performance data from the 2014 Minnesota Community Measurement (MNCM) Health Care Quality Report (http://mncm.org/health-care-quality-report/).

## Appendix

**Table 2:** Measures publicly-available within the MNCM dataset

Performance Domain	Measure
Quality	Breast Cancer Screening
Quality	Colorectal Cancer Screening
Quality	Diabetes
Quality	Vascular Disease
Patient Experience	Patient Experience / Courteous and helpful office staff
Patient Experience	Patient Experience / How well providers communicate
Patient Experience	Patient Experience / Providers with a most positive rating
Cost	Total Cost: Overall
Access	Patient Experience / Getting care when needed
Quality (measures not selected^a^)	ADHD
	Adolescent Immunization
	Asthma - Adults
	Asthma - Children
	Bronchitis
	COPD
	Cervical Cancer Screening
	Childhood Immunization
	Chlamydia Screening
	Colds
	Depression - PHQ-9 Follow-up at 12 Months
	Depression - Use of the PHQ-9
	Depression Remission at 12 Months
	Depression Remission at Six Months
	Depression Response at 12 Months
	Depression Response at Six Months
	Depression: Follow Up (6 Months)
	High Blood Pressure
	Maternity - Cesarean Deliveries
	Mental Health Screening: Teens
	Overweight Counseling: Children
	Sore Throats
	Spinal Surgery: Lumbar Discectomy/Laminotomy w/Pre-Op & Post-Op ODI
	Spinal Surgery: Lumbar Fusion w/Pre-Op & Post-Op ODI
Cost (Measures not selected^a^)	Total Cost: Adults
	Total Cost: Pediatrics
	Total Knee Replacement: Assessing Symptoms Before and After Surgery

^a^Represents measures reported within the MNCM dataset but not included in our study because 1) they were not reported by a majority of medical groups and/or 2) they are specialized measures only applicable to small subgroups

## Abbreviations

CAHPS: Consumer Assessment of Healthcare Providers and Systems
CG-CAHPS: Clinician Group Consumer Assessment of Healthcare Providers and Systems
CMS: Centers for Medicare and Medicaid Services
HEDIS: Healthcare Effectiveness Data and Information Set
IHA: Integrated Healthcare Association
IOM: Institute of Medicine
MNCM: Minnesota Community Measurement
NCQA: National Committee on Quality Assurance
